# Supplementary material for: Preparation of long single-strand DNA concatemers for high-level fluorescence in situ hybridization
Source: Commun Biol. 2021 Oct 25;4:1224. doi: 10.1038/s42003-021-02762-2 (PMC8545947; doi:10.1038/s42003-021-02762-2)
Supplement: Supplementary file 2 — Supplementary information. [file 42003_2021_2762_MOESM2_ESM.pdf]

Supplementary Files

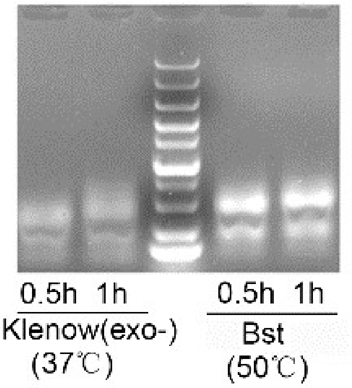

Supplementary Figure 1 The lssDNAcs were assayed by agarose gel electrophoresis. The lssDNAcs were amplified by Klenow (exo-) DNA polymerase (lane 1 for 0.5 h, lane 2 for 1 h) and Bst 2.0 DNA polymerase (lane 3 for 0.5 h, lane 4 for 1 h).

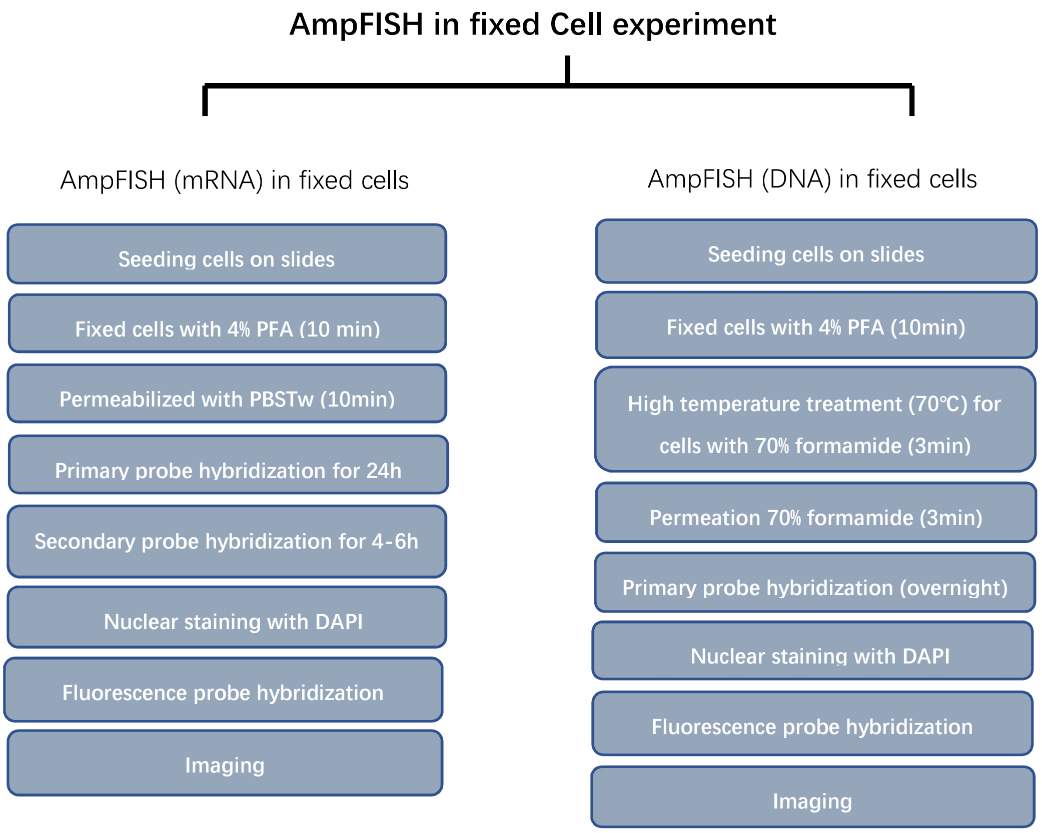

Supplementary Figure 2. Shown is the schematic of the AmpFISH method in fixed cells.

The left panel shows AmpFISH for fixed cells, and the right panel shows DNA FISH for chromosome in fixed cells.

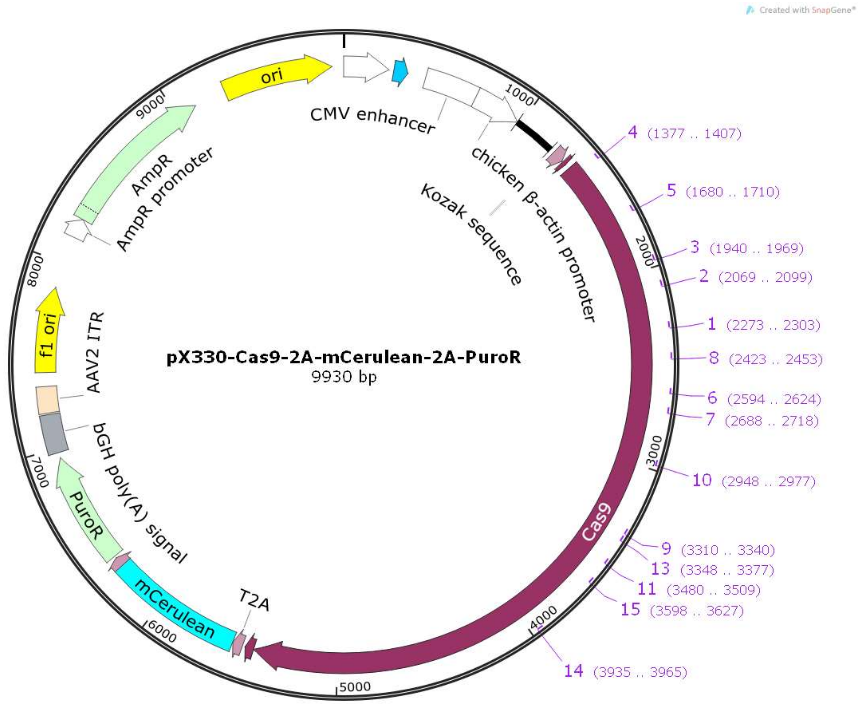

Supplementary Figure 3 The plasmid of pCAG-Cas9-2A-mCerulean-2A-PuroR. The binding sites for lssDNAcs were marked next to Cas9.

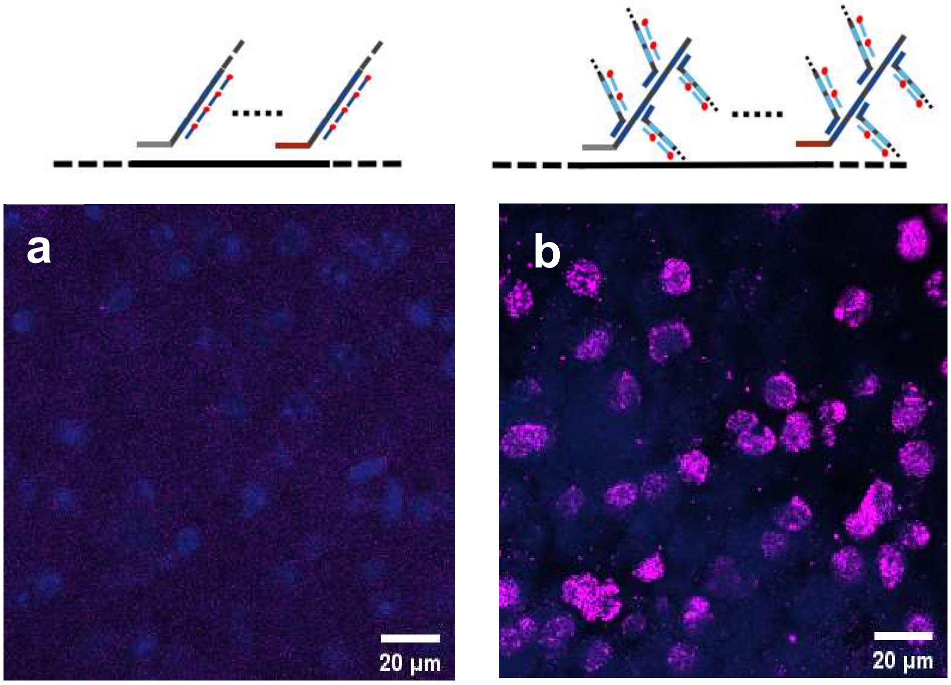

Supplementary Figure 4. AmpFISH imaging with (**a**) primary amplification probes for *Gad1* mRNA in the mouse brain slices and (**b**) secondary amplification probes via branching amplification. Scale bar: 20 μm

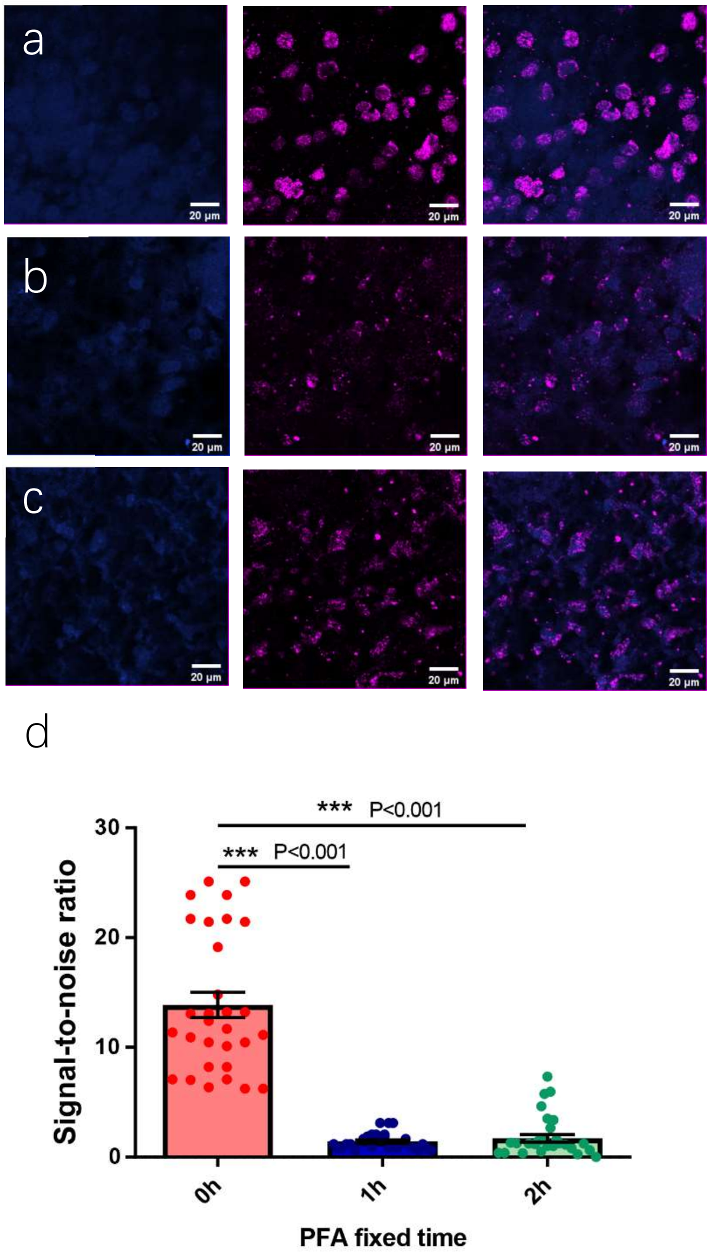

Supplementary Figure 5. Post-fixation with PFA results in a decrease in the signal-to-noise ratio.

During post-fixation, the entire brain tissue was immersed in 4% PFA for further fixation after cardiac perfusion with 4% PFA (see the left panel in Supplementary Figure 5). To determine the effect of post-fixation, the entire brain tissue was extracted from the body and post-fixed for (**a**) 0 h (without treatment), (**b**) 1h, and (**c**) 2h, after the mice were perfused with 4% PFA. Subsequently, the samples of brain slices were hybridized with *Gad1* primary and secondary AmpFISH probes in the mice brains. Scale bar: 20 μm. (**d**) Shown are the results of the ANOVA for the signal-to-noise ratio of the three experiments. ($P < 0.001$, repeated three times).

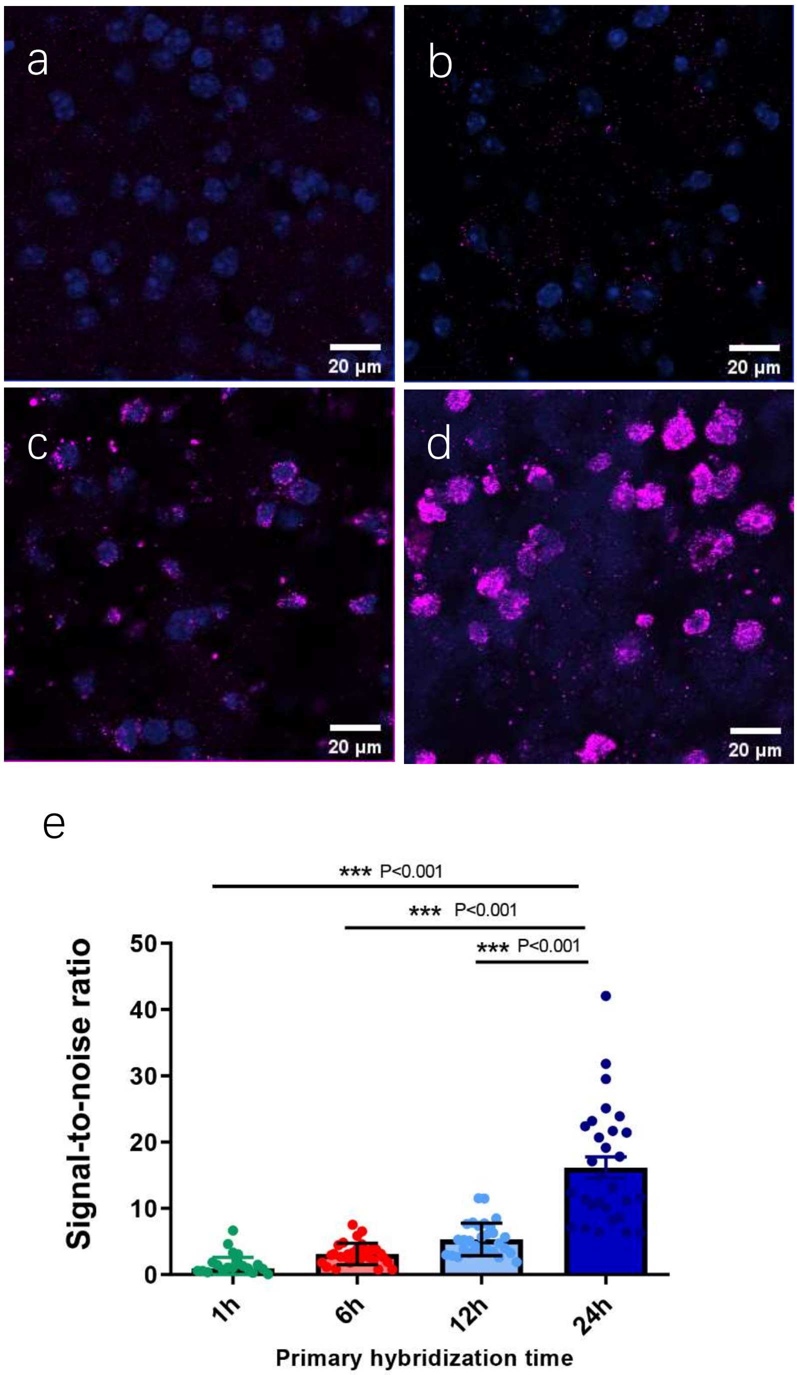

Supplementary Figure 6. The signal-to-noise ratio of AmpFISH was increased by extending the hybridization time.

(**a**)-(**d**) A set of primary amplification probes for *Gad1* mRNA was hybridized for (**a**) 1h, (**b**) 6h, (**c**) 12h, and (**d**) 24h in C57 mouse brain sections with 4% PFA perfusion without post-fixation. Then, all the samples were analyzed using the same protocols and imaging conditions as that shown in the schematic in Supplementary Figure 5. Scale bar: 20 μm (**e**) The signal-to-noise ratios of the four groups in (a)-(d) were compared using ANOVA (repeated three times, $P < 0.001$).

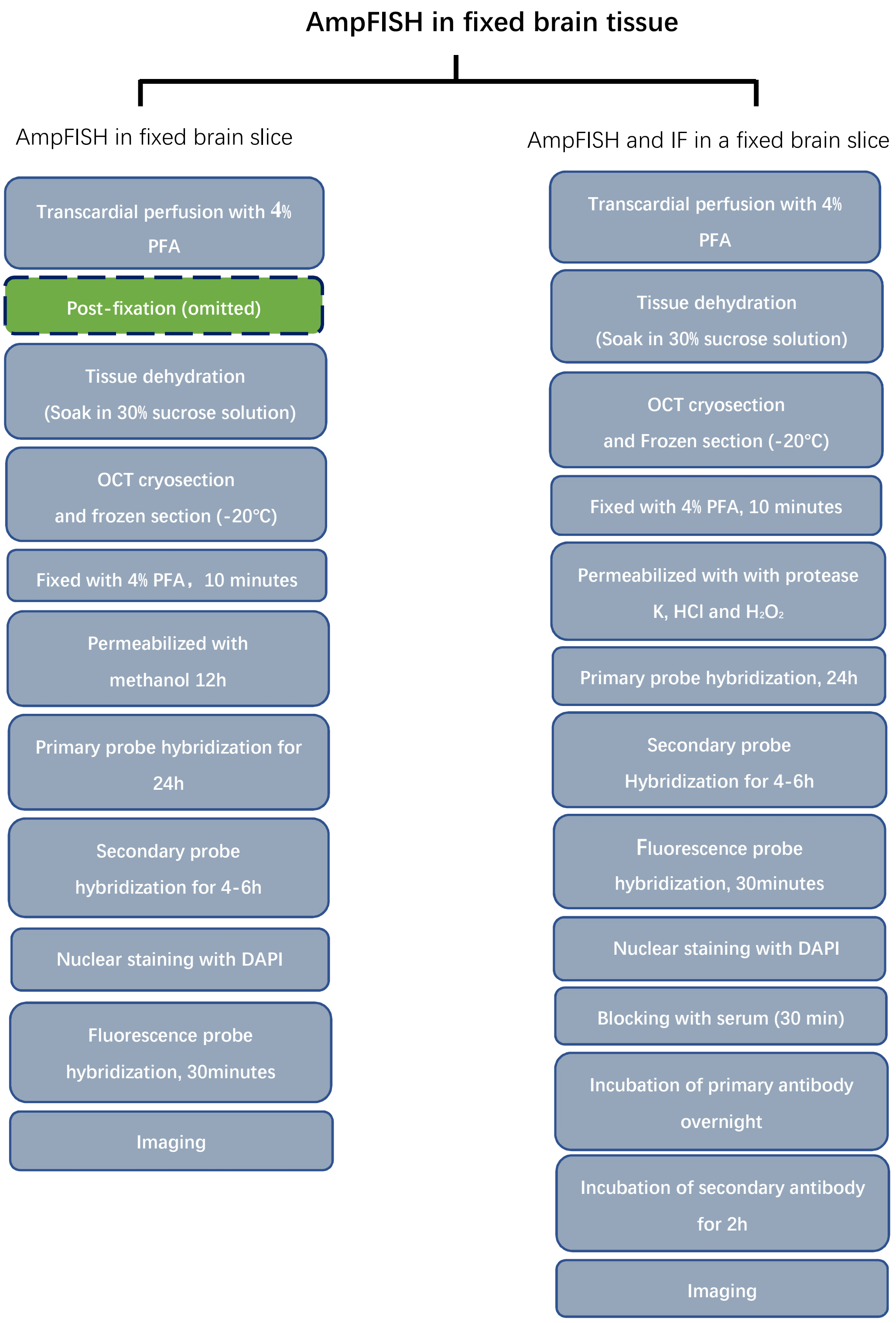

Supplementary Figure 7. Shown is the schematic of the AmpFISH method to brain tissue.

The left panel is for AmpFISH in a fixed brain slice, and the right panel is for AmpFISH with IF in a fixed brain slice.

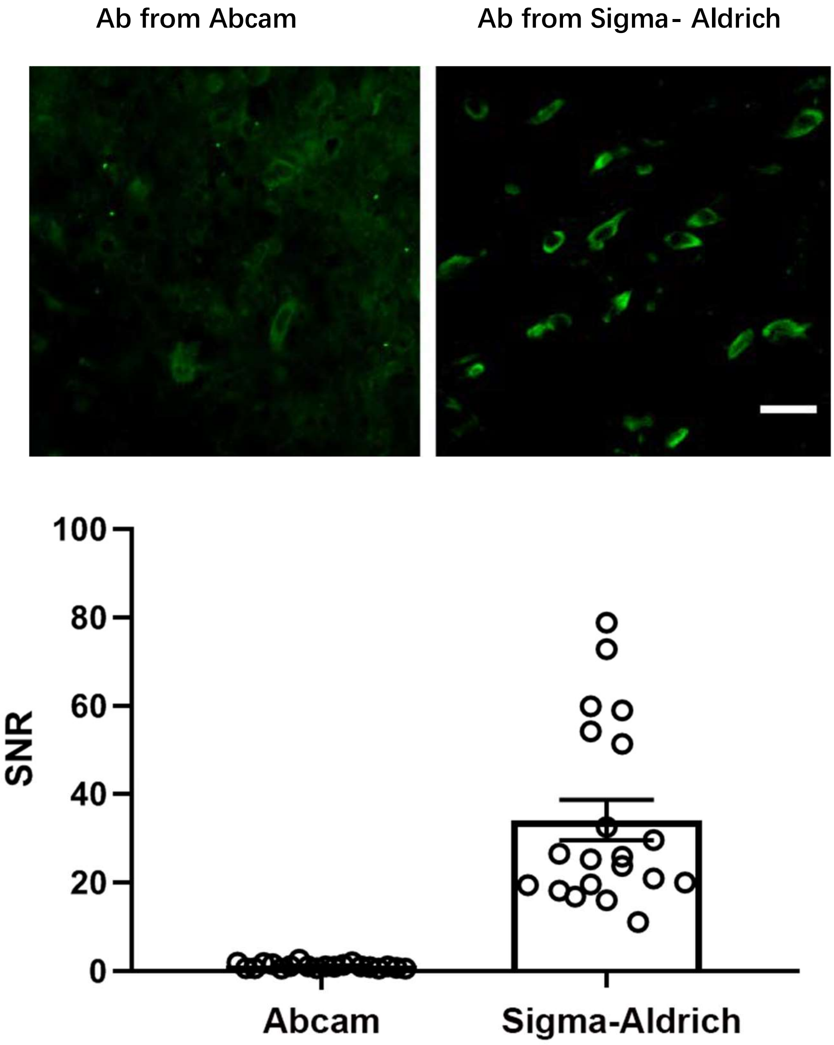

Supplementary Figure 8 The signal-to-noise (SNR) of IF imaging using two anti-Th antibodies from Abcam (No. ab6211) and Sigma-Aldrich (No. AB152) exhibits a significant difference. This indicates IF imaging is greatly dependent on the antibody titer. The antibody titers from different sources vary, resulting in unstable IF signals. Scale bar: 30 μm

Supplementary Note 1

A 200 μL amplification system was used and 1.2 μL and 1.0 μL of amplification primers and ring primers were added to the system, respectively. The average recycled amount of probes ranged from 3 μg to 5 μg. One sample needed a 0.5 μg LssDNAc, that is, one tube of probes (50 μL) can be used in 6 to 10 samples.

Bst 2.0 DNA polymerase: 8000 U/mL, total volume 200 μL, ￥680/tube. A 200 μL amplification system needs 2 μL Bst 2.0 DNA polymerase. The cost for each tube is ￥6.8.

T4 ligase use: 350 U/μL, 25000 U, ￥117 from Takara corp. The cost of each tube of ligase is ￥3.3.

Oligonucleotides synthesis: ￥8 - ￥10 for each amplification reaction.

To prepare lssDNAc probe (50 μL, averaging 60–100 ng/μL) in one tube, ￥3.3 (T4 DNA ligase), ￥6.8 (Bst 2.0 DNA polymerase), and ￥8–10 (oligonucleotides) were required. The total cost was ￥18.1–￥20.1.

Each tube reaction of lssDNAc probes can be used for around 6 to 10 samples, that is, the cost of each lssDNAc probe for each sample is between ￥2.0 and ￥3.0. Each sample need one to five lssDNAcs. So total cost of lssDNAc probes including secondary probes should be ￥2.0–￥15 ($0.28–$2.1) for each sample.

Supplementary Note 2

The sequences of RNA detected in this study are listed, and the binding sequences for the toehold of primary probes are highlighted in yellow.

1. The coding frame of *Cas9-2A-mCerulean-2A-PuroR* and binding sequences for the toehold of primary probes are in red.

ATGGACTATAAGGACCACGACGGAGACTACAAGGATCATGATATTGATTACAAAGACGATGACGATA
AGATGGCCCCAAAGAAGAAGCGGAAGGTCGGTATCCACGGAGTCCCAGCAGCCGACAAGAAGTACA
GCATCGGCCTGGACATCGGCACCAACTCTGTGGGCTGGGCCGTGATCACCGACGAGTACAAGGTGC
CCAGCAAGAAATTCAAGGTGCTGGGCAACACCGACCGGCACAGCATCAAGAAGAACCTGATCGGAG
CCCTGCTGTTCGACAGCGGCGAAACAGCCGAGGCCACCCGGCTGAAGAGAACCGCCAGAAGAAGAT
ACACCAGACGGAAGAACCGGATCTGCTATCTGCAAGAGATCTTCAGCAACGAGATGGCCAAGGTGG
ACGACAGCTTCTTCCACAGACTGGAAGAGTCCTTCCTGGTGGAAGAGGATAAGAAGCACGAGCGGCA
CCCCATCTTCGGCAACATCGTGGACGAGGTGGCCTACCACGAGAAGTACCCCACCATCTACCACCTG
AGAAAGAAACTGGTGGACAGCACCGACAAGGCCGACCTGCGGCTGATCTATCTGGCCCTGGCCCAC
ATGATCAAGTTCCGGGGCCACTTCCTGATCGAGGGCGACCTGAACCCCGACAACAGCGACGTGGAC
AAGCTGTTCATCCAGCTGGTGCAGACCTACAACCAGCTGTTCGAGGAAAACCCCATCAACGCCAGCG
GCGTGGACGCCAAGGCCATCCTGTCTGCCAGACTGAGCAAGAGCAGACGGCTGGAAAATCTGATCG
CCCAGCTGCCCGGCGAGAAGAAGAATGGCCTGTTCGGAAACCTGATTGCCCTGAGCCTGGGCCTGAC
CCCCAACTTCAAGAGCAACTTCGACCTGGCCGAGGATGCCAAACTGCAGCTGAGCAAGGACACCTAC
GACGACGACCTGGACAACCTGCTGGCCCAGATCGGCGACCAGTACGCCGACCTGTTTCTGGCCGCCA
AGAACCTGTCCGACGCCATCCTGCTGAGCGACATCCTGAGAGTGAACACCGAGATCACCAAGGCCCC
CCTGAGCGCCTCTATGATCAAGAGATACGACGAGCACCACCAGGACCTGACCCTGCTGAAAGCTCTC
GTGCGGCAGCAGCTGCCTGAGAAGTACAAAGAGATTTTCTTCGACCAGAGCAAGAACGGCTACGCCG
GCTACATTGACGGCGGAGCCAGCCAGGAAGAGTTCTACAAGTTCATCAAGCCCATCCTGGAAAAGAT
GGACGGCACCGAGGAACTGCTCGTGAAGCTGAACAGAGAGGACCTGCTGCGGAAGCAGCGGACCTT
CGACAACGGCAGCATCCCCCACCAGATCCACCTGGGAGAGCTGCACGCCATTCTGCGGCGGCAGGA
AGATTTTTACCCATTCCTGAAGGACAACCGGGAAAAGATCGAGAAGATCCTGACCTTCCGCATCCCCT
ACTACGTGGGCCCTCTGGCCAGGGGAAACAGCAGATTCGCCTGGATGACCAGAAAGAGCGAGGAAA
CCATCACCCCCTGGAACTTCGAGGAAGTGGTGGACAAGGGCGCTTCCGCCCAGAGCTTCATCGAGCG
GATGACCAACTTCGATAAGAACCTGCCCAACGAGAAGGTGCTGCCCAAGCACAGCCTGCTGTACGAG
TACTTCACCGTGTATAACGAGCTGACCAAAGTGAAATACGTGACCGAGGGAATGAGAAAGCCCGCCT
TCCTGAGCGGCGAGCAGAAAAAGGCCATCGTGGACCTGCTGTTCAAGACCAACCGGAAAGTGACCG
TGAAGCAGCTGAAAGAGGACTACTTCAAGAAAATCGAGTGCTTCGACTCCGTGGAAATCTCCGGCGT
GGAAGATCGGTTCAACGCCTCCCTGGGCACATACCACGATCTGCTGAAAATTATCAAGGACAAGGAC
TTCCTGGACAATGAGGAAAACGAGGACATTCTGGAAGATATCGTGCTGACCCTGACACTGTTTGAGGA
CAGAGAGATGATCGAGGAACGGCTGAAAACCTATGCCCACCTGTTCGACGACAAAGTGATGAAGCA
GCTGAAGCGGCGGAGATACACCGGCTGGGGCAGGCTGAGCCGGAAGCTGATCAACGGCATCCGGG
ACAAGCAGTCCGGCAAGACAATCCTGGATTTCCTGAAGTCCGACGGCTTCGCCAACAGAAACTTCAT
GCAGCTGATCCACGACGACAGCCTGACCTTTAAAGAGGACATCCAGAAAGCCCAGGTGTCCGGCCA
GGGCGATAGCCTGCACGAGCACATTGCCAATCTGGCCGGCAGCCCCGCCATTAAGAAGGGCATCCT
GCAGACAGTGAAGGTGGTGGACGAGCTCGTGAAAGTGATGGGCCGGCACAAGCCCGAGAACATCGT
GATCGAAATGGCCAGAGAGAACCAGACCACCCAGAAGGGACAGAAGAACAGCCGCGAGAGAATGA
AGCGGATCGAAGAGGGCATCAAAGAGCTGGGCAGCCAGATCCTGAAAGAACACCCCGTGGAAAACA
CCCAGCTGCAGAACGAGAAGCTGTACCTGTACTACCTGCAGAATGGGCGGGATATGTACGTGGACCA
GGAACTGGACATCAACCGGCTGTCCGACTACGATGTGGACCATATCGTGCCTCAGAGCTTTCTGAAG
GACGACTCCATCGACAACAAGGTGCTGACCAGAAGCGACAAGAACCGGGGCAAGAGCGACAACGT

GCCCTCCGAAGAGGTCGTGAAGAAGATGAAGAACTACTGGCGGCAGCTGCTGAACGCCAAGCTGAT
TACCCAGAGAAAGTTCGACAATCTGACCAAGGCCGAGAGAGGCGGCCTGAGCGAACTGGATAAGGC
CGGCTTCATCAAGAGACAGCTGGTGGAAACCCGGCAGATCACAAAGCACGTGGCACAGATCCTGGA
CTCCCGGATGAACACTAAGTACGACGAGAATGACAAGCTGATCCGGGAAGTGAAAGTGATCACCCTG
AAGTCCAAGCTGGTGTCCGATTTCCGGAAGGATTTCCAGTTTTACAAAGTGCGCGAGATCAACAACTA
CCACCACGCCCACGACGCCTACCTGAACGCCGTCGTGGGAACCGCCCTGATCAAAAAGTACCCTAA
GCTGGAAAGCGAGTTCGTGTACGGCGACTACAAGGTGTACGACGTGCGGAAGATGATCGCCAAGAG
CGAGCAGGAAATCGGCAAGGCTACCGCCAAGTACTTCTTCTACAGCAACATCATGAACTTTTTCAAGA
CCGAGATTACCCTGGCCAACGGCGAGATCCGGAAGCGGCCTCTGATCGAGACAAACGGCGAAACCG
GGGAGATCGTGTGGGATAAGGGCCGGGATTTTGCCACCGTGCGGAAAGTGCTGAGCATGCCCCAAGT
GAATATCGTGAAAAAGACCGAGGTGCAGACAGGCGGCTTCAGCAAAGAGTCTATCCTGCCCAAGAG
GAACAGCGATAAGCTGATCGCCAGAAAGAAGGACTGGGACCCTAAGAAGTACGGCGGCTTCGACAG
CCCCACCGTGGCCTATTCTGTGCTGGTGGTGGCCAAAGTGGAAAAGGGCAAGTCCAAGAAACTGAAG
AGTGTGAAAGAGCTGCTGGGGATCACCATCATGGAAAGAAGCAGCTTCGAGAAGAATCCCATCGACT
TTCTGGAAGCCAAGGGCTACAAAGAAGTGAAAAAGGACCTGATCATCAAGCTGCCTAAGTACTCCCT
GTTCGAGCTGGAAAACGGCCGGAAGAGAATGCTGGCCTCTGCCGGCGAACTGCAGAAGGGAAACGA
ACTGGCCCTGCCCTCCAAATATGTGAACTTCCTGTACCTGGCCAGCCACTATGAGAAGCTGAAGGGCT
CCCCCGAGGATAATGAGCAGAAACAGCTGTTTGTGGAACAGCACAAGCACTACCTGGACGAGATCAT
CGAGCAGATCAGCGAGTTCTCCAAGAGAGTGATCCTGGCCGACGCTAATCTGGACAAAGTGCTGTCC
GCCTACAACAAGCACCGGGATAAGCCCATCAGAGAGCAGGCCGAGAATATCATCCACCTGTTTACCC
TGACCAATCTGGGAGCCCCTGCCGCCTTCAAGTACTTTGACACCACCATCGACCGGAAGAGGTACAC
CAGCACCAAAGAGGTGCTGGACGCCACCCTGATCCACCAGAGCATCACCGGCCTGTACGAGACACG
GATCGACCTGTCTCAGCTGGGAGGCGACAAAAGGCCGGCGGCCACGAAAAAGGCCGGCCAGGCAA
AAAAGAAAAAGGAATTCGGCAGTGGAGAGGGCAGAGGAAGTCTGCTAACATGCGGTGACGTCGAGG
AGAATCCTGGCCCCATGGTGAGCAAGGGCGAGGAGCTGTTCACCGGGGTGGTGCCCATCCTGGTCG
AGCTGGACGGCGACGTAAACGGCCACAAGTTCAGCGTGTCCGGCGAGGGCGAGGGCGATGCCACCT
ACGGCAAGCTGACCCTGAAGTTCATCTGCACCACCGGCAAGCTGCCCGTGCCCTGGCCCACCCTCGT
GACCACCCTGACCTGGGGCGTGCAGTGCTTCGCCCGCTACCCCGACCACATGAAGCAGCACGACTTC
TTCAAGTCCGCCATGCCCGAAGGCTACGTCCAGGAGCGCACCATCTTCTTCAAGGACGACGGCAACT
ACAAGACCCGCGCCGAGGTGAAGTTCGAGGGCGACACCCTGGTGAACCGCATCGAGCTGAAGGGCA
TCGACTTCAAGGAGGACGGCAACATCCTGGGGCACAAGCTGGAGTACAACGCCATCAGCGACAACG
TCTATATCACCGCCGACAAGCAGAAGAACGGCATCAAGGCCAACTTCAAGATCCGCCACAACATCGA
GGACGGCAGCGTGCAGCTCGCCGACCACTACCAGCAGAACACCCCCATCGGCGACGGCCCCGTGCT
GCTGCCCGACAACCACTACCTGAGCACCCAGTCCAAGCTGAGCAAAGACCCCAACGAGAAGCGCGA
TCACATGGTCCTGCTGGAGTTCGTGACCGCCGCCGGGATCACTCTCGGCATGGACGAGCTGTACAAG
GAGGGCAGAGGAAGTCTTCTAACATGCGGTGACGTGGAGGAGAATCCCGGCCCTAGGCTCGAGATG
ACCGAGTACAAGCCCACGGTGCGCCTCGCCACCCGCGACGACGTCCCCAGGGCCGTACGCACCCTC
GCCGCCGCGTTCGCCGACTACCCCGCCACGCGCCACACCGTCGATCCGGACCGCCACATCGAGCGG
GTCACCGAGCTGCAAGAACTCTTCCTCACGCGCGTCGGGCTCGACATCGGCAAGGTGTGGGTCGCGG
ACGACGGCGCCGCGGTGGCGGTCTGGACCACGCCGGAGAGCGTCGAAGCGGGGGCGGTGTTCGCC
GAGATCGGCCCGCGCATGGCCGAGTTGAGCGGTTCCCGGCTGGCCGCGCAGCAACAGATGGAAGGC
CTCCTGGCGCCGCACCGGCCCAAGGAGCCCGCGTGGTTCCTGGCCACCGTCGGCGTCTCGCCCGAC
CACCAGGGCAAGGGTCTGGGCAGCGCCGTCGTGCTCCCCGGAGTGGAGGCGGCCGAGCGCGCCGG
GGTGCCCGCCTTCCTGGAGACCTCCGCGCCCCGCAACCTCCCCTTCTACGAGCGGCTCGGCTTCACC
GTCACCGCCGACGTCGAGGTGCCCGAAGGACCGCGCACCTGGTGCATGACCCGCAAGCCCGGTGCC
TGA

2. *EYFP* sequence and binding sequences for the toehold of primary probes are in red.

ATGGTGAGCAAGGGCGAGGAGCTGTTCACCGGGGTGGTGCCCATCCTGGTCGAGCTGGACGGCGA
CGTAAACGGCCACAAGTTCAGCGTGTCCGGCGAGGGCGAGGGCGATGCCACCTACGGCAAGCTGA
CCCTGAAGTTCATCTGCACCACCGGCAAGCTGCCCGTGCCCTGGCCCACCCTCGTGACCACCTTCGG
CTACGGCCTGCAGTGCTTCGCCCGCTACCCCGACCACATGAAGCAGCACGACTTCTTCAAGTCCGCC
ATGCCCGAAGGCTACGTCCAGGAGCGCACCATCTTCTTCAAGGACGACGGCAACTACAAGACCCGC
GCCGAGGTGAAGTTCGAGGGCGACACCCTGGTGAACCGCATCGAGCTGAAGGGCATCGACTTCAAG
GAGGACGGCAACATCCTGGGGCACAAGCTGGAGTACAACTACAACAGCCACAACGTCTATATCATG
GCCGACAAGCAGAAGAACGGCATCAAGGTGAACTTCAAGATCCGCCACAACATCGAGGACGGCAG
CGTGCAGCTCGCCGACCACTACCAGCAGAACACCCCCATCGGCGACGGCCCCGTGCTGCTGCCCGA
CAACCACTACCTGAGCTACCAGTCCGCCCTGAGCAAAGACCCCAACGAGAAGCGCGATCACATGGT
CCTGCTGGAGTTCGTGACCGCCGCCGGGATCACTCTCGGCATGGACGAGCTGTACAAGTAA

3. *EGFP* sequence and binding sequences for the toehold of primary probes are in red.

ATGGTGAGCAAGGGCGAGGAGCTGTTCACCGGGGTGGTGCCCATCCTGGTCGAGCTGGACGGCGA
CGTAAACGGCCACAAGTTCAGCGTGTCCGGCGAGGGCGAGGGCGATGCCACCTACGGCAAGCTGA
CCCTGAAGTTCATCTGCACCACCGGCAAGCTGCCCGTGCCCTGGCCCACCCTCGTGACCACCCTGAC
CTACGGCGTGCAGTGCTTCAGCCGCTACCCCGACCACATGAAGCAGCACGACTTCTTCAAGTCCGCC
ATGCCCGAAGGCTACGTCCAGGAGCGCACCATCTTCTTCAAGGACGACGGCAACTACAAGACCCGC
GCCGAGGTGAAGTTCGAGGGCGACACCCTGGTGAACCGCATCGAGCTGAAGGGCATCGACTTCAAG
GAGGACGGCAACATCCTGGGGCACAAGCTGGAGTACAACTACAACAGCCACAACGTCTATATCATG
GCCGACAAGCAGAAGAACGGCATCAAGGTGAACTTCAAGATCCGCCACAACATCGAGGACGGCAG
CGTGCAGCTCGCCGACCACTACCAGCAGAACACCCCCATCGGCGACGGCCCCGTGCTGCTGCCCGA
CAACCACTACCTGAGCACCCAGTCCGCCCTGAGCAAAGACCCCAACGAGAAGCGCGATCACATGGT
CCTGCTGGAGTTCGTGACCGCCGCCGGGATCACTCTCGGCATGGACGAGCTGTACAAG

4. Mouse *Gad1* sequence (a part of whole sequence) and binding sequences for the toehold of primary probes are in red.

CAGCGCTCCGCGGACGAGCTGCCCCCGCGAGCAACGGCCTCGTGATTCCCCCCGCCGAGCGGGTCC
CCGCCTCCCCACTCCGCCCCCGCCTCCCCCAAGCCCAGCGGCCGCCTCTCCGGATCTCTCCCTTCTT
CAGGCTCTCCCGTGCCGGACCAGGGATCGTGCAAGCAAGGAAGCAGCCCTGGGGTGACACCCAGC
ACGTACTCCTGTGACAGAGCCGAGCCCAGCCCAGCCCCGGGACGCTTCGCAGAGGAGTCGCGGGA
GGGTCCAGCTCGCTGTCGCTGAACCGAGCCTGTTCCTGCGCCCAGTCTGCGGGGGACCCTTGAACC
GTAGAGACCCCAAGACCACCGAGCTGATGGCATCTTCCACTCCTTCGCCTGCAACCTCCTCGAACGC
GGGAGCGGATCCTAATACTACCAACCTGCGCCCTACAACGTATGATACTTGGTGTGGCGTAGCCCAT
GGATGCACCAGAAAACTGGGCCTGAAGATCTGTGGCTTCTTACAAAGGACCAATAGCCTGGAAGAG
AAGAGTCGTCTTGTGAGCGCCTTCAGGGAGAGGCAGTCCTCCAAGAACCTGCTTTCCTGTGAAAACA
GTGACCAGGGTGCCCGCTTCCGGCGCACAGAGACCGACTTCTCCAACCTGTTTGCTCAAGATCTGCT
TCCAGCTAAGAACGGGGAGGAGCAAACTGCGCAGTTCTTGCTGGAAGTGGTAGACATACTCCTCAA
CTATGTCCGCAAGACATTTGATCGCTCCACCAAGGTTCTGGATTTCCACCACCCACACCAGTTGCTGG
AAGGCATGGAAGGCTTTAATTTGGAGCTGTCTGACCACCCCGAGTCTCTGGAGCAGATCCTGGTTGA
CTGTAGAGACACCCTGAAGTACGGGGTTCGCACAGGTCACCCTCGATTTTTCAACCAGCTCTCTACTG
GTTTGGATATCATTGGTTTAGCTGGTGAATGGCTGACATCGACTGCCAATACCAATATG

5. Mouse *CaMKIIα* sequence (a part of whole sequence) and binding sequences for the toehold of primary probes are in red.

TATGCTGGCCACCAGGAACTTCTCCGGAGGGAAGAGCGGAGGAAACAAGAAGAACGATGGTGTGA

AGGAATCTTCTGAGAGCACCAACACCACCATTGAGGACGAAGACACCAAAGTGCGCAAACAGGAAA
TTATCAAAGTGACAGAGCAGCTGATCGAAGCCATAAGCAATGGAGACTTTGAGTCCTACACGAAGAT
GTGCGACCCTGGAATGACAGCCTTTGAACCAGAGGCCCTGGGGAACCTGGTGGAGGGCCTGGACTT
TCATCGATTCTATTTTGAAAACCTGTGGTCCCGGAACAGCAAGCCCGTGCACACCACCATCCTGAAC
CCTCACATCCACCTGATGGGTGACGAGTCAGCCTGCATCGCCTATATCCGCATCACTCAGTACCTGG
ATGCAGGCGGCATACCCCGCACGGCCCAGTCAGAGGAGACCCGCGTCTGGCACCGCAGGGACGGC
AAATGGCAGATCGTCCACTTCCACAGATCTGGGGCGCCCTCCGTCCTGCCGCATTGAAGGACCAGG
CCAGGGTCCCTGCGTCCTTGCTTCGCAGAGATCCGCTCTTTGTCCGTGGAATGTGGCTGCTGGTTCTC
CTTTGGATTTTGCTGGAATTCTCCCTGTCAGATCACCCTACCATTGCCACCTATGTACTCGCGTCACGA
AAACCTGCTTGTTCACAGAAGTCGCCACGACATCACAGTGAACAGCCAGCTCTCCCCAGCTCCGTTG
CCCAAGCTCTTCCTGCCAGTGGGGACCTTCTTCCGGCTTAAGTACCCAGGGTGCTGGCCCCAGGAAC
CCCCACCCCCTACCCACTGTTGTTGGCCTAGCCTAGCTTTAGCTATAGATGGGGCCTCAGCTGTGCAA
TTGGCAGGAAGTGAGGAAGAGGCAGGCAAGCTGTGTTGAGGGCACCTCTCATCGATTCCTTCTTTCC
TGGGGTTCCCCGGGGAAGCTCACACGAGGCCCTCAGTCTCCAAGCCAACCCCTTATGAGGGAGAGT
GAGAGAGGAGCCAACGCCAGTGAGCCAGGAACTGCTGCTCTCATCTGCTCTCCTCTGTGTTGGCCTT
GCCTTTGACCAGACCATCCGCTACGAGGGGTGGGCTCTACCGCCCAGGTGCCCCACTCACTCTGCCT

6. Mouse *Actinβ* sequence (a part of whole sequence) and binding sequences for the toehold of primary probes are in red.

GTTCGCCATGGATGACGATATCGCTGCGCTGGTCGTCGACAACGGCTCCGGCATGTGCAAAGCCGG
CTTCGCGGGCGACGATGCTCCCCGGGCTGTATTCCCCTCCATCGTGGGCCGCCCTAGGCACCAGGGT
GTGATGGTGGGAATGGGTCAGAAGGACTCCTATGTGGGTGACGAGGCCCAGAGCAAGAGAGGTATC
CTGACCCTGAAGTACCCCATTGAACATGGCATTGTTACCAACTGGGACGACATGGAGAAGATCTGGC
ACCACACCTTCTACAATGAGCTGCGTGTGGCCCCTGAGGAGCACCCTGTGCTGCTCACCGAGGCCCC
CCTGAACCCTAAGGCCAACCGTGAAAAGATGACCCAGAGATCATGTTTGAGACCTTCAACACCCCAG
CCATGTACGTAGCCATCCAGGCTGTGCTGTCCCTGTATGCCTCTGGTCGTACCACAGGCATTGTGATG
GACTCCGGAGACGGGGTCACCCACACTGTGCCCATCTACGAGGGCTATGCTCTCCCTCACGCCATCC
TGCGTCTGGACCTGGCTGGCCGGGACCTGACAGACTACCTCATGAAGATCCTGACCGAGCGTGGCT
ACAGCTTCACCACCACAGCTGAGAGGGAAATCGTGCGTGACATCAAAGAGAAGCTGTGCTATGTTG
CTCTAGACTTCGAGCAGGAGATGGCCACTGCCGCATCCTCTTCCTCCCTGGAGAAGAGCTATGAGCT
GCCTGACGGCCAGGTCATCACTATTG

7. *Lox* sequence and binding sequences for the toehold of primary probes are in red.

CACGTCCTCCCCGAGAAGGGACGAGCCGGGAGCACCATGCGTTTCGCCTGGGCTGTGCTCCTTCTG
GGGCCACTGCAGCTTTGTCCCCTTCTCCGCTGCGCCCCGCAGACCCCGCGCGAGCCGCCCGCCGCC
CCTGGTGCCTGGCGCCAGACAATCCAATGGGAGAACAACGGGCAGGTGTTCAGTCTGCTGAGCCTC
GGGGCGCAGTACCAGCCTCAGCGACGCCGCGACCCCAGTGCCACTGCCCGGAGACCCGACGGCGA
CGCAGCCTCGCAGCCGCGCACGCCCATTCTTCTGCTGCGTGACAACCGCACCGCCTCTACCCGTGCG
AGGACGCCAAGCCCGTCTGGGGTCGCCGCGGGTCGTCCCCGGCCCGCCGCCCGCCACTGGTTCCAA
GCTGGTTTCTCGCCGTCGGGGGCTCGCGATGGAGCCTCACGGCGCGCGGCGAACCGGACTGCATCG
CCACAGCCTCCGCAGCTCAGTAATCTGAGGCCACCCAGCCACATAGATCGCATGGTGGGCGACGAC
CCCTACAATCCCTACAAGTACTCCGACGACAACCCCTATTATAACTACTATGACACGTATGAGAGGC
CCCGGCCCGGGAGCAGGAACCGACCTGGATACGGCACCGGTTACTTCCAGTACGGTCTCCCGGACC
TGGTGCCCGACCCCTACTACATCCAGGCTTCCACGTACGTCCAGAAGATGTCTATGTACAACCTGAG
ATGCGCTGCGGAAGAAAACTGCCTGGCCAGTTCAGCATATAGGGCGGATGTCAGAGACTATGACCA
CAGGGTACTGCTACGATTTCCGCAAAGAGTGAAGAACCAAGGGACATCGGACTTCTTACCAAGCCG
CCCTCGGTACTCCTGGGAGTGGCACAGCTGTCACCAACATTACCACAGCATGGACGAATTCAGCCAC
TATGACCTGCTTGATGCCAACACACAGAGGAGAGTGGCTGAAGGCCACAAAGCAAGCTTCTGTCTG

GAGGACACGTCCTGTGACTATGGGTACCACAGGCGCTTTGCGTGCACTGCACACACACAGGGATTG
AGTCCTGGATGTTATGACACCTATGCGGCAGACATAGACTGCCAGTGGATTGATATTACAGATGTAC
AACCTGGAAACTACATTCTAAAGGTCAGTGTAAACCCCAGCTACCTGGTGCCTGAATCAGACTACAC
TAACAATGTTGTACGCTGTGACATTCGCTACACAGGACATCATGCCTATGCCTCAGGCTGCACAATTT
CACCGTATTAGAAAGAAGCCCAGTTCCCAGTGGATAAAGCCGTACCTGGTGTTTGGACATATGGAAA
CTATAGATTAGCTTAAGTAGGAAGGACTTACATATTTTAAAAGACAAACAGTAAACAACAAAGGAGG
TTTTGTTTGGACTCTTTCGCAACAAATGACATAACTGGATTTTGAGTGTTTAAACCAACAATATTTGGC
AAATTTTAAATCCTTACTCATGTTATTTTGTGAATTAGCAGTGTTTCAATTCTGTGGGTGCATAGTGGG
CTCTTTCAAAGAACTCTGAATTTCTTATGTTCTTTTGAAATTATAGTGCAAATGAGAAATTGATATTTTA
ATGAGTGAGCCACAATTTGAACGGATTCTTTTCTAAAGTGCTGAAACCAGTGAAACAATGATGATGA
GCCTATATTTGCCTCGACTTAGATTGATTTTTTAAAAAGGTGTCCCTATTGTGTAAACAGAAATGGATA
CACTTGGTGCTGAGGAAGGGCCAAACATCTAACTATTGTTGTCATGAAGTATAATCAGAAAGATGGC
GATATATGTATTCAGATAGTTACATCCCTATATAAAATTATGTTTACATTTTAGAGTTACTAGACAATTT
CCTTTCTCTTAATTGCTTAATTTCACTCTGACTTGAGTCAACTTGTTTTGGAATGAATTGGAAAATTCCA
AGATCCTGTTTGATACTTGTTGCTATTCGATCCCACGCTGCTTAGCTTTTCTGTGGGCAGAAATGTCTA
ATGTGACAATCAGCACATCCCCATTGTGAGGTTTCACGGCATAGGGAAATCATTCCGATGACCAACA
GCTGTGATCAGTGGAGAGCTAACATGCCCTGCAGCATATTTTACTGTTAGCCAGAATTAAGTCACTGC
TGACACAGCAATTATACAAGGAATCTCTAACATCACAATCATCTTTCAAATACTGTCAAAAGAAAATA
CTGGAAAACCTTTTAGATTTTTTTTTCAACTCTACTACACTTCTGAAATGTTCAGCTATTAAGGAGAAT
GTTGAATTTATATATATATAAAACCATCCGTAATAGATGACCTACTGTGTGCAATGCAGCACCATCAT
CTTAGACTCTTCAGGAGTTGGGTATATACGTTTTTGTGTATTTACAAAATACAACCCAAATTAGCGAAG
CACATAGCATTGTATACTTGAGAGGTTGGCGAACAAGAGGGAAGCAGAGCCTTCCTGCAAAAACCA
AGGAGCACGCACCACAACGAGACCGCTGTGACCTTACGTAAAACTGTGAAACTGTGTGTAGCAGTG
GGATTTGGAGACTCTTACATTCAGAAGGTGCAGAAAGAAATCAAGTTAGACTTTTAAATTCCTGGTGC
TTGTGCCGAAGTCGTACTTCATTTACCAGCGTTAAAATCTCAAGTAAGGCATCTCCTCCTGTGGTAGA
ACGTGTCAGGTAGAGCTTTCTAAGTGAGCTGGGATCCTGACTCACGTGTCATAGACTTGTAAGAGCA
GCAGTCTCTAATCATCTCGCTGTAGACTCATTGCTGCAGAGCAAGTGGGAGGGGCCAGAGATGGGC
AGTCGCCAGGGGTGGATAGCGCGGTACACATAATTCTCATCTGCCTGAAAGCACACTTACCCATAAG
CAAATGCAGGCAGAACTCTGATTGAGCTGGTTTCTGATCCACAGGATTGCTTCTTCGATGTACCTCTTT
GCCCTAGCTAGCATTTCAGTTTTGATACTGTAGAACGATCCTTTCAAATTATAATCATTCTGATAGAGG
TATTTTAATATACATGCTTTTAAAAACAAAACAAAAAACTACTGTCAGTATGAATACTGAGCCAGACT
GGCATATATAGATTTAACATCTTGTCCTACTAAGATTCTTAACTGTATAAAAATAATATGGCTTTTAGA
CATATAGGATACTAATTTCAATGAGACCCTTATCTCTTTATTGAACATTATGTTAGGGACAGTAAAAGC
CATGCACTTACCTGCTACCCATTGG

8. *Txn1* sequence and binding sequences for the toehold of primary probes are in red.

CCAGGCGCCTTGGCGGACGCGGGAGGCGGGAAGCGCGTCCCCGCCGCGCGTGCCCGTCCGCCCCG
CCCTGCTGCGCTCCGCCCTATTTCTATAAAGAGAAGGCGGGCGCCGCACTCGGTTGCAAGCTCCGTT
GGGCGCCTTGGATCCATTTCCATCTGGTTCTGCTGAGACGCGTGTGGCTCCCT/CCCCGCAACAGCCA
AAATGGTGAAGCTGATCGAGAGCAAGGAAGCTTTTCAGGAGGCCCTGGCCGCCGCGGGAGACAAG
CTTGTCGTGGTGGACTTCTCTGCTACGTGGTGTGGACCTTGCAAAATGATCAAGCCCTTCTTCCATTCC
CTCTGTGACAAGTATTCCAATGTGGTGTTCCTTGAAGTGGATGTGGATGACTGCCAGGATGTTGCTGC
AGACTGTGAAGTCAAATGCATGCCGACCTTCCAGTTTTATAAAAAGGGTCAAAAGGTGGGGGAGTTC
TCCGGTGCTAACAAGGAAAAGCTTGAAGCCTCTATTACTGAATATGCCTAATCATGCTCTGAAAAGTG
TAACCAGCTACCAGCTGTTTAAAACCTGTACCTTTTTTAATTTGCAAAAAACTATGAAGTGTGGAGAG
TCTATACCCAACTGCCATCTGATTATAAATGACAATAAAATATTAATTCTACCCTTTTTAAAACTGCCT
GATGTGTTTTAATATAAATGAGAAATGGCCTGATTTAGTCCCCTAAGCCACTGCTTTAAGGCAAAAAG
ATAGGAGCTGGAGAGGTGGCTCAGTCGTTTAGAACATTGCCTGTTCTTGCAGAGGGCCAAAGTTCAG
TTCTCAGCATCCATACGGCAGCTACCTGTTCCTCCAGTTCCAGGGAATCTGATGTCCTCTACTGGCCA

CCATGGGCACCAAGCATGTCTGAAGTACATGTACATTACATGTAACTAACATACACATAAACTTTTTA
AAATCTTTTTTTAAAACTTAATATTTAGTTGTAGAAAATCTCGTTTATTCGGTTACATTAAAAGTCTTGG
GGGGGGGGTATTTTACCTTAATAAATAAACATGGTCC

9. *Sla2* sequence and binding sequences for the toehold of primary probes are in red.

AGTAGCACCTGGGTCTGTCTACACGGGCTCAGGTCCCTAGGCCACGCTCTTTGTCCCTGCTGTGCTG
GGTGGGAGGGCCCAATCTGGTTTCTCTGAGAAGCAAAGGACTGCTGTACTAGTTTCGTGGAGATTGT
CTGCTGACAAAGAAGCTTGATCACAGTACCTCAGCCTACTCTGACTCCTTTCTGGTGACCGATCCTCC
AGGCTGCTGGGGCCTGAGATGCCGACTACCTTAGGACCTGCAAAGGCCTGACCTGTCGGGTCAGTG
TGCACATTGGCTGACTACCCTCATCAAACGTCTGATGGCAAACCTTTCCCTTTCCAGGTTCAGTGTGC
TTGTGAGCGTCTGCTGAGTGATGGGAAGTTTGTCCAGCAGAGGGAAAACCTCCAGCCCCAGCCCCA
GCTCCTCTGGTCCAGACCAGGAACCCGTGTCCATGCAACCAGAAAGACACAAGGTCACAGCTGTGG
CCCTGGGCAGTTTCCCAGCAGGTGAACAGGCCAGACTATCTCTGAGACTCGGGGAGCCGCTGACCA
TCATCTCTGAGGATGGAGATTGGTGGACAGTCCAGTCGGAAGTCTCAGGCAGAGAGTACCACATGC
CCAGTGTGTATGTGGCTAAAGTCGCCCACGGGTGGCTGTACGAGGGCCTGAGCCGGGAGAAAGCCG
AGGAACTACTCCTGTTACCTGGGAACCCCGGAGGGGCCTTCCTCATCCGGGAGAGCCAGACCAGGA
GAGGCTGCTATTCCCTGTCCGTCCGACTCAGCCGCCCTGCATCTTGGGACCGGATCAGACACTACAG
GATACAGCGTCTTGACAATGGCTGGCTGTACATCTCACCTCGCCTCACCTTCCCCTCACTCCACGCCT
TGGTGGAGCATTACTCTGAGCTAGCAGATGGCATCTGCTGTCCCCTCAGGGAGCCGTGTGTCCTGCA
GAAGCTTGGGCCACTACCTGGCAAAGATACACCTCCACCTGTGACTGTGCCAACATCATCACTAAAT
TGGAAAAAGCTGGACCGCAGCCTCCTGTTTCTGGAAGCACCTGCGAGTGGGGAGGCATCTCTGCTCA
GTGAGGGGCTCCGAGAGTCCCTCAGTTCCTACATCAGCCTGGCTGAGGACCCCTTGGATGATGCTTA
GCCCTGGAGCACAAAGAGAAAGGGAACCAAGACTGTGGCACCGAGAGCTCCAACTCCGCTGACCC
TGACAAAGCTCCAGGAGGCAAGGCTGGGAGAACAGAGACGGCTGGGGTGGGGCACAGACACTCGG
GGCCTCACCTGGGCTTTCTGATAAGTCATGTATCTCCTAAGGCCTCACCCCTACCTACTACTTCTAGTC
CATGTGCAGTGCAGTTCAAAGCAGGGCTGGCCTCTACAGAGAATAAAATACTTCTGAGGTCTGATAA
GTCACTTCCTGAGAGAAATGTCCAGTACAGCAGCTACTCAGCCCTACTAAAAGACAGAGAGAGCATC
ACTACCATGGAGAGAAAAAATATTGGGAACGCTCACAAGTCTTTTCCAGAATGCAAACACATCTCAG
ATGGTAGGATGGCTCAGAAAGCATTGACAAGAGGAACACTGGATGGCTTTACCGCAGTAGACCCCA
CCTAACTCCCACGCACGAGGAAGACTGACAATCCAGATCTTCCCATGATCTCCCATCAAGTCCTTTCA
GCCAACTTCAGTGTTCAAACAGCCAGGTCCTCCCCATACCCCCGTTTTGCCACCTGGTGGTCAAACA
CAAAATGACACCGTTTAAATCTCACATCTAGCCCAGTCACCTATCTCAGTACTGAGTCAGGACATCAG
GTGGTGGACCCTCTCTGAAATGCCAATACCAACTCCAGCCAAAAGAGAAAGAGTCTGGCTGCTACTG
CTGCTGTGGGAGGAGAGGGAGGAGGGAAGAGAGCAGGGGCTCTGCTAAACCCTACTCCTAGGTTC
AGAGGGTAAGGTCAGTAGGTAAGCAAGGCTTTAGTTTACCAGGCATGCCAACACACTTTCAGTTAAA
GATTTGTTTATTCAAGTTTCTGAAAACATCCTACAATGGAGACTTATTAGATGGCTGCTTGTATTGTAC
CATGGACCACACACATGGTGTTTTCAGAAGACTTGAGATGCAGTGATGGTTTAAAAACTGTACACCT
GACGGAGAACCAAGGAAGACAATGTTTCATCCGGATCCAGAGGCGCATCAAATTCAACAGCAGCTC
CACTCGGCAAACAGCTGTTACATGATGGTATCCAAGGAAAAACAGTTGACAATGGACAAATTCAGAA
ATGCTTCCCCAAAGGCGGATGGTTCTTTGAAAAGTTTCACAACCCTTGCGGAAAATACTGATGTCCAA
CACGTTGATTCTAAGTTCAGGAGACGTGGGTCTTCCCGGTTCCACGCGGCAGGAGTGCCCCAATTGG
CATGTACCTGTGCTGTAGTAAAAGGGTCCTTCGGACTGGACAGAAGACACGGGAGCAAATGCCATC
AGTCATAGTGGATGGCAGTGTAAAAGATGATCCAAATGACCTACGGGAGACACAAGCAATATTCATA
TCGTCACCTGGATCAACACAGGCTCCTCCCCCACATGAAGGCAGCAATGTATAAATCTGAGGTCTTT
AGGATGTTCATAAAGGATAATTATCAT

Supplementary Table. Shown are the oligo sequences that were used in this study.

**Fig.2a oligo synthesis**	
EYFP-1	ATGAACTTCAGGGTCAGCTTGCCGTAGGTGGCATCG TTAGTTGGGATGTATTGAAGGAGGAT
EYFP-2	TTGAAGAAGATGGTGCGCTCCTGGACGTAGCCTTCG TTAGTTGGGATGTATTGAAGGAGGAT
EYFP-3	TAGTTGTACTCCAGCTTGTGCCCCAGGATGTTGCCG TTAGTTGGGATGTATTGAAGGAGGAT
EYFP-4	TGGCGGATCTTGAAGTTCACCTTGATGCCGTTCTTC TTAGTTGGGATGTATTGAAGGAGGAT
Padlock for EYFP	p-AATACATCCCAACTAAACCCTCTAACTTCCATCACAACAGTAATCCTCCTTC
Fluo-EYFP	ACCCTCTAACTTCCATCACA(Cy5)
**Fig.2b oligo synthesis: AmpFISH for *Actinβ*, *Lox*, *Txn*, and *Sla2***	
Lox-P1	AGGTCACAGCGGTCTCGTTGTGGTGCGTGCTCCTTGGT TTAGTTGGGATGTATT GAAGGAGGAT
Lox-P2	ATGTGGCTGGGTGGCCTCAGATTACTGAGCTGCGGAGGCT TTAGTTGGGATGTATT GAAGGAGGAT
Lox-P3	TGCCACTCCCAGGAGTACCGAGGGCGGCTTGGTAAGAAGT TTAGTTGGGATGTATT GAAGGAGGAT
Lox-P4	TGCACGCAAAGCGCCTGTGGTACCCATAGTCACAGGACGT TTAGTTGGGATGTATT GAAGGAGGAT
Lox-P5	AGGCTCAGCAGACTGAACACCTGCCCGTTGTTCTCCCAT TTAGTTGGGATGTATT GAAGGAGGAT
Txn-P1	ATCAGCTTCACCATTTTGGCTGTTGCGGGGAGGGAGCCAC TTAGTTGGGATGTATT GAAGGAGGAT
Txn-P2	TGCGGCGCCCGCCTTCTCTTTATAGAAATAGGGCGGAGCG TTAGTTGGGATGTATT GAAGGAGGAT
Txn-P3	AGGGAGCCACACGCGTCTCAGCAGAACCAGATGGAAATGG TTAGTTGGGATGTATT GAAGGAGGAT
Txn-P4	ACATGCTTGGTGCCCATGGTGGCCAGTAGAGGACATCAGA TTAGTTGGGATGTATT GAAGGAGGAT
Txn-P5	CCCTGGAACTGGAGGAACAGGTAGCTGCCGTATGGATGCT TTAGTTGGGATGTATT GAAGGAGGAT
Actinβ-P1	ACAGCACAGCCTGGATGGCTACGTACATGGCTGGGGTGT TTAGTTGGGATGTATT GAAGGAGGAT
Actinβ-P2	AGCTCTTCTCCAGGGAGGAAGAGGATGCGGCAGTGGCCAT TTAGTTGGGATGTATT GAAGGAGGAT
Actinβ-P3	TGGGTGACCCCGTCTCCGGAGTCCATCACAATGCCTGT TTAGTTGGGATGTATT GAAGGAGGAT
Actinβ-P4	AGCCGTTGTCGACGACCAGCGCAGCGATATCGTCATCCAT TTAGTTGGGATGTATT GAAGGAGGAT
Actinβ-P5	CAGCTGTGGTGGTGAAGCTGTAGCCACGCTCGGTCAGGAT TTAGTTGGGATGTATT GAAGGAGGAT
Sla2-P1	AGCCACCCGTGGGCGACTTTAGCCACATACACACTGGGCA TTAGTTGGGATGTATT GAAGGAGGAT
Sla2-P2	TCAGGGTCAGCGGAGTTGGAGCTCTCGGTGCCACAGTCTT TTAGTTGGGATGTATT GAAGGAGGAT
Sla2-P3	AGTCAGCCAATGTGCACACTGACCCGACAGGTCAGGCCT TTAGTTGGGATGTATT GAAGGAGGAT
Sla2-P4	ACAGGTACATGCCAATTGGGGCACTCCTGCCGCGT TTAGTTGGGATGTATT GAAGGAGGAT
Sla2-P5	ACTGAGCAGAGATGCCTCCCCACTCGCAGGTGCTTCCAGA TTAGTTGGGATGTATT GAAGGAGGAT
Padlock for *Actinβ*, *Lox*, *Txn*, *Sla2* 1st amplification: p-AATACATCCCAACTAA ACCCTCTAACTTCCATCACAACAGTA ATCCTCCTTC	
The second round hybridization for *Actinβ*, *Lox*, *Txn*, *Sla2*	
Padlock-2nd	pTTCAAGTCTTTCCTAT TCTCAACCTAACCTTCCTATTCTCAACCTA ACCTTCGAGT
2nd-adaptor	ACCCTCTAACTTCCATCACAACAGTAATCCTCCTTC ATAGGAAAGACTTGAA ACTCGAAGGT
Fluor-2nd-Tamra	TCCTATTCTCAACCTAACCT(TAMRA)
Padlock-Lox	pAATACATCCCAACTAA ACCCTCTAACTTCCATCACAACAGTA ATCCTCCTTC

**Fig.2d oligo synthesis: smFISH for *Cas9***	
Cas9 -1	AGGCGCTCAGGGGGGCCTTGGTGATCTCGGT ATAGGAAATGGTGGTAGTGT
Cas9 -2	AGTTGGGGGTCAGGCCCAGGCTCAGGGCAAT ATAGGAAATGGTGGTAGTGT
Cas9 -3	GGCGTCCACGCCGCTGGCGTTGATGGGGTT ATAGGAAATGGTGGTAGTGT
Cas9 -4	ATCACGGCCCAGCCCACAGAGTTGGTGCCGA ATAGGAAATGGTGGTAGTGT

Cas9 -5	ATGTTGCCGAAGATGGGGTGCCGCTCGTGCT ATAGGAAATGGTGGTAGTGT
Cas9 -6	CCTGCCGCCGCAGAATGGCGTGCAGCTCTCC ATAGGAAATGGTGGTAGTGT
Cas9 -7	CCCCTGGCCAGAGGGCCCACGTAGTAGGGGA ATAGGAAATGGTGGTAGTGT
Cas9 -8	CCTGGCTGGCTCCGCCGTCAATGTAGCCGGC ATAGGAAATGGTGGTAGTGT
Cas9-9	CTTCCGGCTCAGCCTGCCCCAGCCGGTGT ATATAGGAAATGGTGGTAGTGT
Cas9-10	CTGCTCGCCGCTCAGGAAGGCGGGCTTTCT ATAGGAAATGGTGGTAGTGT
Cas9-11	CGTGCAGGCTATCGCCCTGGCCGGACACCT ATAGGAAATGGTGGTAGTGT
Cas9-12	CCAGTTCGCTCAGGCCGCCTCTCTCGGCCT ATAGGAAATGGTGGTAGTGT
Cas9-13	TCTTGCCGGACTGCTTGTCCCGGATGCCGT ATAGGAAATGGTGGTAGTGT
Cas9-14	CTTCGGAGGGCACGTTGTCGCTCTTGCCCCG ATAGGAAATGGTGGTAGTGT
Cas9-15	ATCACGATGTTCTCGGGCTTGTGCCGGCCC ATAGGAAATGGTGGTAGTGT
Fluor-Tamra (Cas9)	ACACTACCAC CATTTCCTAT(TAMRA)
**Fig.2d oligo synthesis: AmpFISH for *Cas9***	
Cas9 –Amp1	AGGCGCTCAGGGGGGCCTTGGTGATCTCGGTTTAGTTGGGATGTATT GAAGGAGGAT
Cas9 -Amp 2	AGTTGGGGGTCAGGCCCAGGCTCAGGGCAAT TTAGTTGGGATGTATT GAAGGAGGAT
Cas9 -Amp 3	GGCGTCCACGCCGCTGGCGTTGATGGGGTT TTAGTTGGGATGTATT GAAGGAGGAT
Cas9 -Amp4	ATCACGGCCCAGCCCACAGAGTTGGTGCCGA TTAGTTGGGATGTATT GAAGGAGGAT
Cas9 –Amp5	ATGTTGCCGAAGATGGGGTGCCGCTCGTGCT TTAGTTGGGATGTATT GAAGGAGGAT
Cas9 –Amp6	CCTGCCGCCGCAGAATGGCGTGCAGCTCTCC TTAGTTGGGATGTATT GAAGGAGGAT
Cas9 –Amp7	CCCCTGGCCAGAGGGCCCACGTAGTAGGGGA TTAGTTGGGATGTATT GAAGGAGGAT
Cas9 –Amp8	CCTGGCTGGCTCCGCCGTCAATGTAGCCGGC TTAGTTGGGATGTATT GAAGGAGGAT
Cas9 –Amp9	CTTCCGGCTCAGCCTGCCCCAGCCGGTGTAT TTAGTTGGGATGTATT GAAGGAGGAT
Cas9 –Amp10	CTGCTCGCCGCTCAGGAAGGCGGGCTTTCT TTAGTTGGGATGTATT GAAGGAGGAT
Cas9 –Amp11	CGTGCAGGCTATCGCCCTGGCCGGACACCT TTAGTTGGGATGTATT GAAGGAGGAT
Cas9 –Amp12	CCAGTTCGCTCAGGCCGCCTCTCTCGGCCT TTAGTTGGGATGTATT GAAGGAGGAT
Cas9 –Amp13	TCTTGCCGGACTGCTTGTCCCGGATGCCGT TTAGTTGGGATGTATT GAAGGAGGAT
Cas9 –Amp14	CTTCGGAGGGCACGTTGTCGCTCTTGCCCCG TTAGTTGGGATGTATT GAAGGAGGAT
Cas9 –Amp15	ATCACGATGTTCTCGGGCTTGTGCCGGCCC TTAGTTGGGATGTATT GAAGGAGGAT
Padlock-1st-Cas9	p-AATACATCCCAACTAA ACCCTCTAACTTCCATCACAACAGTA ATCCTCCTTC
Fluor-1st-Tamra (Cas9)	ACCCTCTAACTTCCATCACA(TAMRA)
For the second round hybridization	
Cas9-2nd-adaptor	ACCCTCTAACTTCCATCACA GCATGGGATCGTTGT CTGCTTGAGG
Padlock-2nd-Cas9	p-ACAACGATCCCATGCACTCACACCTC AAA ATCCCATGCACTCACACCTCAAGCAG
Fluor-2nd-Tamra (Cas9)	ATCCCATGCACTCACACCTC(TAMRA)
For the third round hybridization	
Adaptor-3rd	ATCCCATGCACTCACACCTC TAGGATAGACTT CTGACTAGGT
Padlock-3rd-Cas9	p-AAGTCTATCCTA TTCTCAACCTAACCTAAATCCTATTCTCAACCTA ACCTAGTCAG
Fluor-3rd-Tamra (Cas9)	TCCTATTCTCAACCTAACCT(TAMRA)
**Oligo synthesis for Fig.3b, Supplementary Figure 3, 7, 8**	
Gad1-P1	CTCGGCGGGGGGAATCACGAGGCCGTTGCT TTAGTTGGGATGTATTGAAGGAGGAT

Gad1-P2	TGCTTGCACGATCCCTGGTCCGGCACGGG TTAGTTGGGATGTATTGAAGGAGGAT
Gad1-P3	CGCTCCCGCGTTCGAGGAGGTTGCAGGCGA TTAGTTGGGATGTATTGAAGGAGGAT
Gad1-P4	TCTGGTGCATCCATGGGCTACGCCACACCA TTAGTTGGGATGTATTGAAGGAGGAT
Gad1-P5	AGGAGTGGAAGATGCCATCAGCTCGGTGGT TTAGTTGGGATGTATTGAAGGAGGAT
Padlock-Gad1	p AATACATCCCAACTAA ACCCTCTAACTTCCATCACAACAGTA ATCCTCCTTC
For the second round hybridization	
2nd-adaptor	ACCCTCTAACTTCCATCACAACAGTAATCCTCCTTC ATAGGAAAGACTTGAA ACTCGAAGGT
Padlock-2nd	p-TTCAAGTCTTTCCTAT TCTCAACCTAACCTTCCTATTCTCAACCTA ACCTTCGAGT
Fluor-2nd-Tamra	TCCTATTCTCAACCTAACCT(TAMRA)
**Fig.3d oligo synthesis**	
EGFP-P1	TCCCGGCGGCGGTCACGAACTCCAGCAGGA GACATCATAGGAAATG GTGGTAGTGT
EGFP-P2	AGCGGCTGAAGCACTGCACGCCGTAGGTCA GACATCATAGGAAATGGTGGTAGTGT
EGFP-P3	GCTCGATGCGGTTCACCAGGGTGTCGCCCT GACATCATAGGAAATGGTGGTAGTGT
EGFP-P4	ACCTCGGCGCGGGTCTTGTAGTTGCCGT GACATCATAGGAAATGGTGGTAGTGT
EGFP-P5	CCTGGACGTAGCCTTCGGGCATGGCGGACT GACATCATAGGAAATGGTGGTAGTGT
Padlock for EYFP	p-CATTTCCTATGATGTC AGAGTGAGTAGTAGTGGAGT ACACTACCAC
For the second round hybridization	
2nd-P1	ACACTACCACCATTTCCTAT TGCATGTGAAAGGAAT GGGTTGTGGT
2nd-P2	ACTCCACTACTACTCACTCT TGCATGTGAAAGGAAT GGGTTGTGGT
Padlock for-2nd	p-ATTCCTTTCACATGCA TTTCTACCACTAATCAACCC ACCACAACCC
Fluro-EYFP	TTTCTACCACTAATCAACCC(TAMRA)
**Fig.4 oligo synthesis**	
Ch8	ATGTATCTACTCAACTAACAGTGCTGAACATTTCTATTGATTAGTTGGGATGTATTGAAGGAGGAT
Padlock-for Ch8	p-AATACATCCCAACTAA ACCCTCTAACTTCCATCACAACAGTA ATCCTCCTTC
Fluo-Ch8	TCCCAACTAAACCCTCTAACTTCCATCACAACAG(TAMRA)
**Fig.5 oligo synthesis**	
CaMKIIα-P1	ACGATCTGCCATTTGCCGTCCCTGCGGTGC TTAGTTGGGATGTATT GAAGGAGGAT
CaMKIIα-P2	GCGGGGTATGCCGCCTGCATCCAGGTACTG TTAGTTGGGATGTATT GAAGGAGGAT
CaMKIIα-P3	AGGCGATGCAGGCTGACTCGTCACCCAT TTAGTTGGGATGTATT GAAGGAGGAT
CaMKIIα-P4	AGCAGCCACATTCCACGGACAAAGAGCGG TTAGTTGGGATGTATT GAAGGAGGAT
CaMKIIα-P5	TGTCGTGGCGACTTCTGTGAACAAGCAGGT TTAGTTGGGATGTATT GAAGGAGGAT
Padlock-CaMKIIα	pAATACATCCCAACTAA ACCCTCTAACTTCCATCACAACAGTA ATCCTCCTTC
The second round hybridization for *CaMKIIα*	
2nd-adaptor	ACCCTCTAACTTCCATCACAACAGTAATCCTCCTTC ATAGGAAAGACTTGAA ACTCGAAGGT
Padlock-2nd	pTTCAAGTCTTTCCTAT TCTCAACCTAACCTTCCTATTCTCAACCTA ACCTTCGAGT
Fluor-2nd-Tamra	TCCTATTCTCAACCTAACCT(TAMRA)
