# Supplementary material for: Preparation of long single-strand DNA concatemers for high-level fluorescence in situ hybridization
Source: Commun Biol. 2021 Oct 25;4:1224. doi: 10.1038/s42003-021-02762-2 (PMC8545947; doi:10.1038/s42003-021-02762-2)
Supplement: Supplementary file 3 — Description of Additional Supplementary Files. [file 42003_2021_2762_MOESM3_ESM.pdf]

Description of Additional Supplementary Files

File name: Supplementary Movie 1
Description: 3D tomography of the cell nucleus of the centromere of chromosome 8 in HeLa cells using AmpFISH.
