# Supplementary material for: Preparation of long single-strand DNA concatemers for high-level fluorescence in situ hybridization
Source: Commun Biol. 2021 Oct 25;4:1224. doi: 10.1038/s42003-021-02762-2 (PMC8545947; doi:10.1038/s42003-021-02762-2)
Supplement: Supplementary file 5 — Reporting summary. [file 42003_2021_2762_MOESM5_ESM.pdf]

nature research

Corresponding author(s): Qingming Luo, Jie Yang

Last updated by author(s): Sep 15, 2021

Reporting Summary

Nature Research wishes to improve the reproducibility of the work that we publish. This form provides structure for consistency and transparency in reporting. For further information on Nature Research policies, see our Editorial Policies and the Editorial Policy Checklist.

Statistics

For all statistical analyses, confirm that the following items are present in the figure legend, table legend, main text, or Methods section.

n/a	Confirmed	
☐	☒	The exact sample size (*n*) for each experimental group/condition, given as a discrete number and unit of measurement
☐	☒	A statement on whether measurements were taken from distinct samples or whether the same sample was measured repeatedly
☐	☒	The statistical test(s) used AND whether they are one- or two-sided<br>*Only common tests should be described solely by name; describe more complex techniques in the Methods section.*
☒	☐	A description of all covariates tested
☐	☒	A description of any assumptions or corrections, such as tests of normality and adjustment for multiple comparisons
☒	☐	A full description of the statistical parameters including central tendency (e.g. means) or other basic estimates (e.g. regression coefficient) AND variation (e.g. standard deviation) or associated estimates of uncertainty (e.g. confidence intervals)
☐	☒	For null hypothesis testing, the test statistic (e.g. *F*, *t*, *r*) with confidence intervals, effect sizes, degrees of freedom and *P* value noted<br>*Give P values as exact values whenever suitable.*
☒	☐	For Bayesian analysis, information on the choice of priors and Markov chain Monte Carlo settings
☒	☐	For hierarchical and complex designs, identification of the appropriate level for tests and full reporting of outcomes
☒	☐	Estimates of effect sizes (e.g. Cohen's *d*, Pearson's *r*), indicating how they were calculated

Our web collection on statistics for biologists contains articles on many of the points above.

Software and code

Policy information about availability of computer code

Data collection	Commercial softwares licensed by microscopy companies were utilized: Zeiss Zen2012(for LSM 710),Nikon Ni-E
Data analysis	ImageJ/FIJI software was used to analyze and overlay images as described in the Methods section. NUPACK software was used to design FISH probe. (http://www.nupack.org/partition/new).The signal intensity statistical analysis was performed using GraphPad Prism 5 .

For manuscripts utilizing custom algorithms or software that are central to the research but not yet described in published literature, software must be made available to editors and reviewers. We strongly encourage code deposition in a community repository (e.g. GitHub). See the Nature Research guidelines for submitting code & software for further information.

Data

Policy information about availability of data

All manuscripts must include a data availability statement. This statement should provide the following information, where applicable:

- Accession codes, unique identifiers, or web links for publicly available datasets
- A list of figures that have associated raw data
- A description of any restrictions on data availability

https://figshare.com/s/51ea12e69cbdfca79e09; Sequencing data were deposited in GEO database (GEO accession numbers: GSE181685).

Field-specific reporting

Life sciences study design

All studies must disclose on these points even when the disclosure is negative.

Sample size	We specified the sample size for each experiment, n=3 was chosen as the minimal replicate number. And the staining, imaging and data analysis results were reliably reproduced in those experiments. We determined this to be sufficient owing to low observed variability between samples.
Data exclusions	No data were excluded.
Replication	Experimental results were reliably reproduced.
Randomization	There was no need for randomization in this technique development.
Blinding	Blinding was not possible as experimental conditions were mostly evident from the image data. Quantifications were performed using computational software applied equally to all conditions and replicates for a given probe.

Reporting for specific materials, systems and methods

We require information from authors about some types of materials, experimental systems and methods used in many studies. Here, indicate whether each material, system or method listed is relevant to your study. If you are not sure if a list item applies to your research, read the appropriate section before selecting a response.

Materials & experimental systems

n/a	Involved in the study	
☐	☒	Antibodies
☐	☒	Eukaryotic cell lines
☒	☐	Palaeontology and archaeology
☐	☒	Animals and other organisms
☒	☐	Human research participants
☒	☐	Clinical data
☒	☐	Dual use research of concern

Methods

n/a	Involved in the study	
☒	☐	ChIP-seq
☒	☐	Flow cytometry
☒	☐	MRI-based neuroimaging

Antibodies

Antibodies used	Primary antibody for CaMKIIa was purchased from GeneTex Inc. (No. GTX127939).<br>Anti-Th antibody was purchased from Sigma-Aldrich (No. AB152) and Abcam (No. ab6211).The secondary antibody is goat anti-rabbit Alexa 488.
Validation	Certificates of analysis (No. GTX127939), (No. AB152),(No. ab6211) can be found on company's website.

Eukaryotic cell lines

Policy information about cell lines

Cell line source(s)	HeLa cells and NIH3T3 cells
Authentication	None of the cell lines have been authenticated.
Mycoplasma contamination	Cell lines were not tested for mycoplasma contamination but no indication of contamination was observed.
Commonly misidentified lines (See ICLAC register)	No commonly misidentified cell lines were used.

Animals and other organisms

Policy information about studies involving animals; ARRIVE guidelines recommended for reporting animal research

Laboratory animals	C57BL/6 mice and Thy1-GFP mice were used in this work.
Wild animals	No wild animals were used in this study.
Field-collected samples	No field-collected samples were used in this study.
Ethics oversight	All animal experiments were approved by the Animal Ethics Committee of Huazhong University of Science and Technology.

Note that full information on the approval of the study protocol must also be provided in the manuscript.
